# Supplementary material for: Risk Factors for Salmonella Detection in Commercial Layer Flocks in Spain
Source: Animals (Basel). 2023 Oct 12;13(20):3181. doi: 10.3390/ani13203181 (PMC10603648; doi:10.3390/ani13203181)

Figure S1. Number of samplings conducted in each bimester (1: Jan-Feb, 2: Mar-Apr, 3: May-Jun, 4: Jul-Aug, 5: Sep-Oct, 6: Nov-Dec).

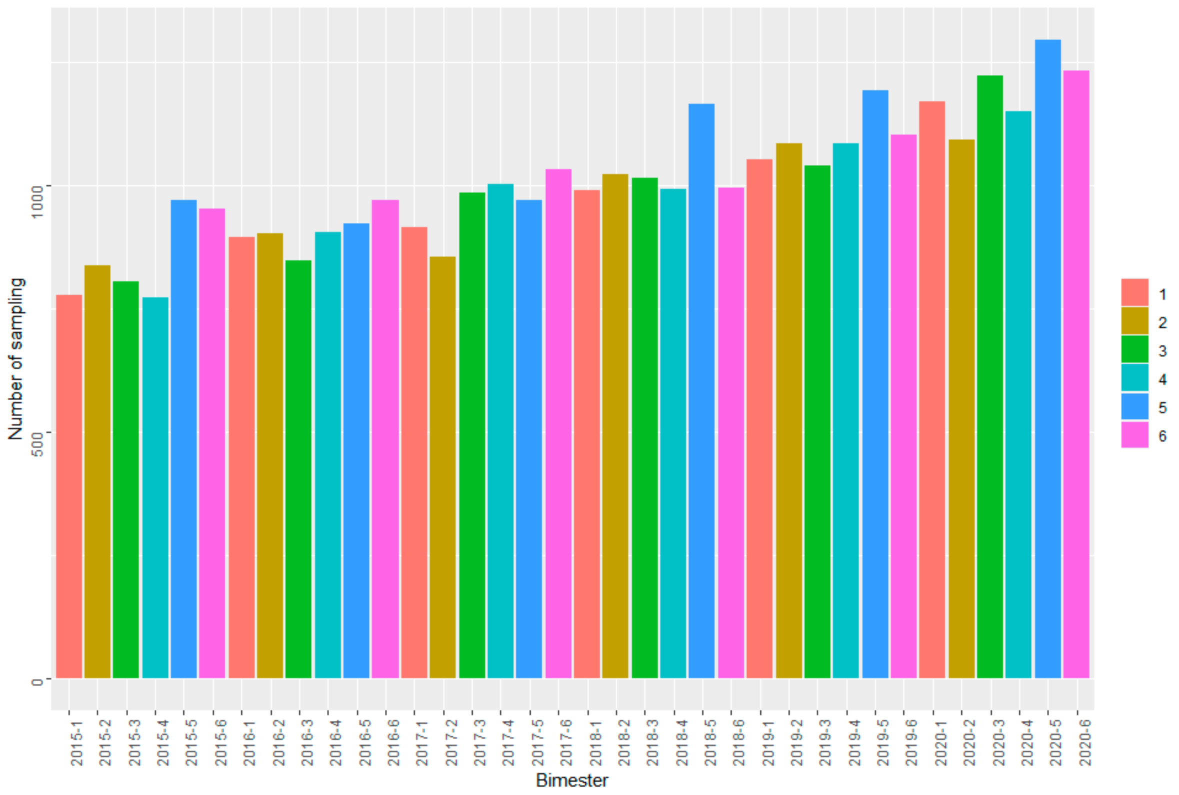

Figure S2. Monthly percentage of positive sampling events depending on the sampler and type over the study period (FBOp: food business operator; CA: competent authority; Env: environmental; rout: routine).

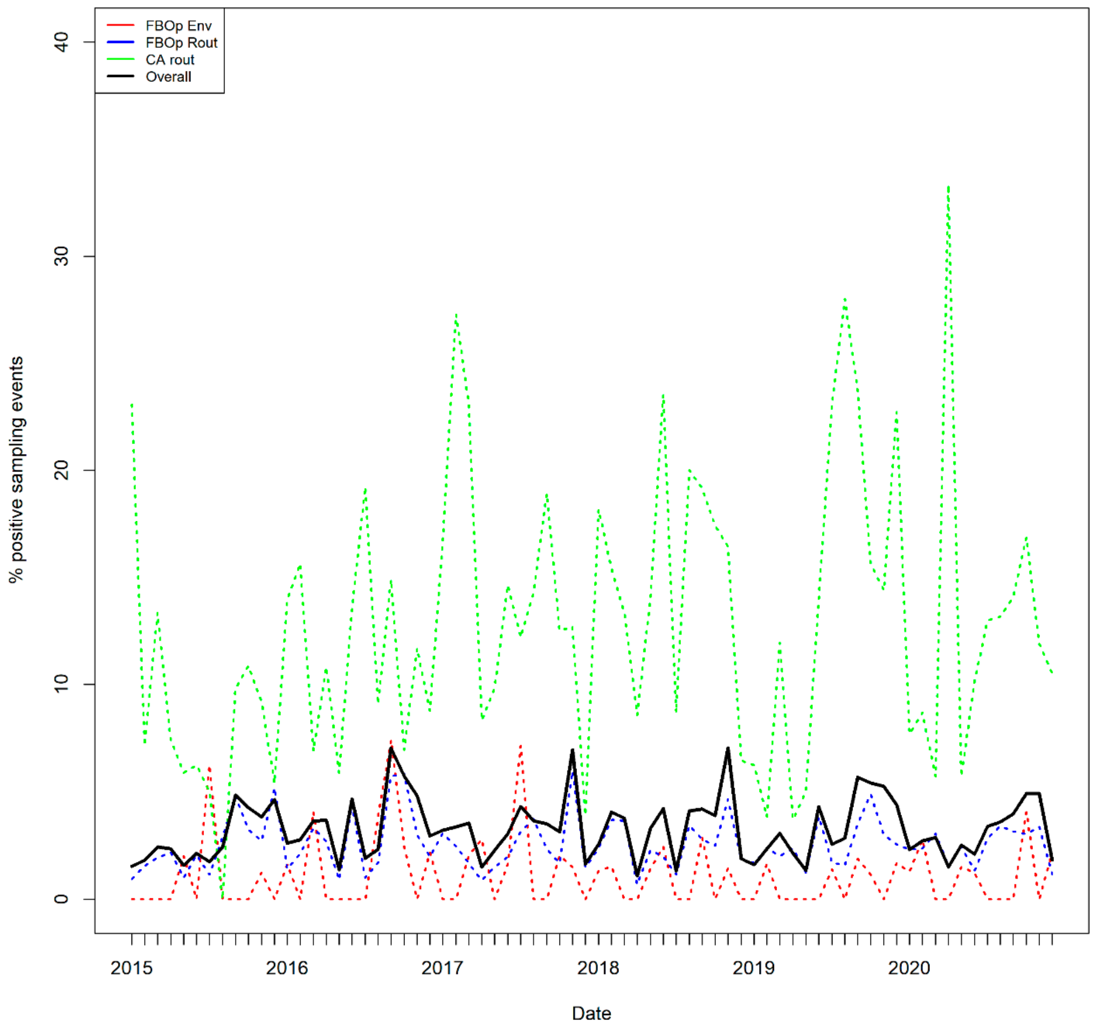

Figure S3. Correlation between proportion of farms/flocks with one or more positive samplings for Salmonella spp. (Farm_pos and Flock_pos) or for target serovars (Farm_target and Flock_target) at the Autonomous Region level.

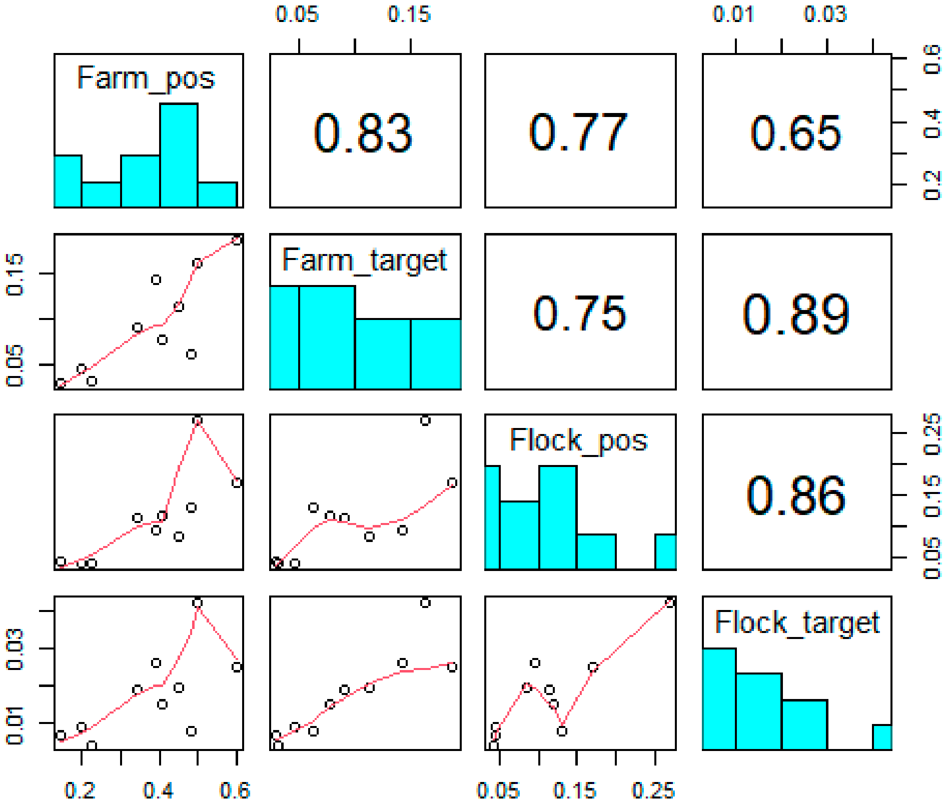

Figure S4. Median estimates for the farm-level random effects (α, n=1154) depending on the region where the farm was located.

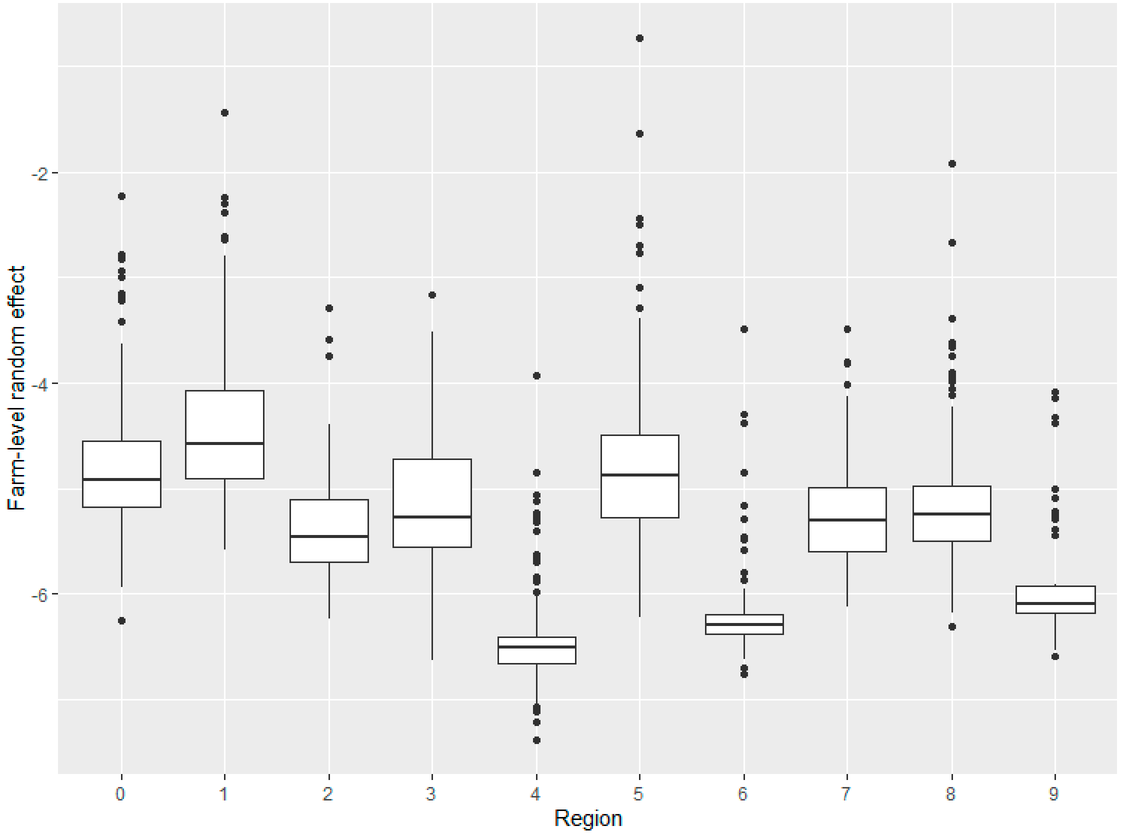

Figure S5. Median estimates for the flock-level random effects (U, n=7219) depending on the region where the flock was located.

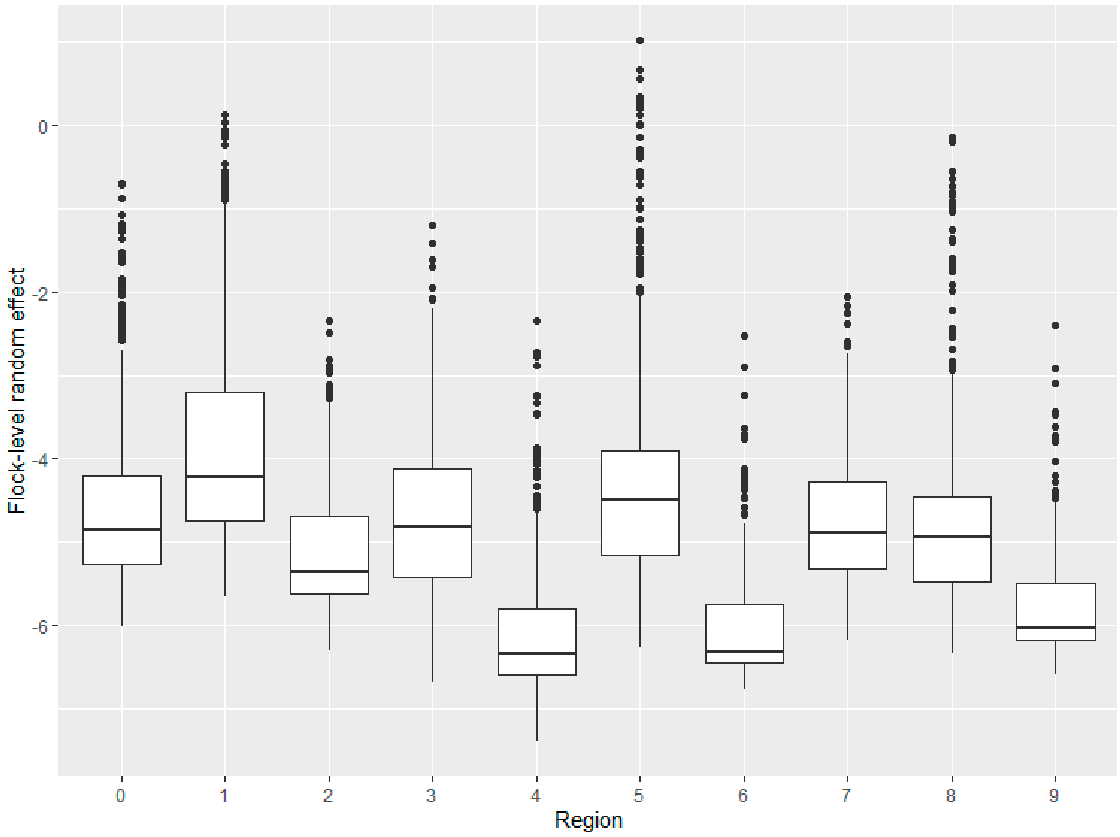

Figure S6. ROC (A) and precision-recall (B) curves based on the probability of a positive result in a sampling for Salmonella (any serovar)

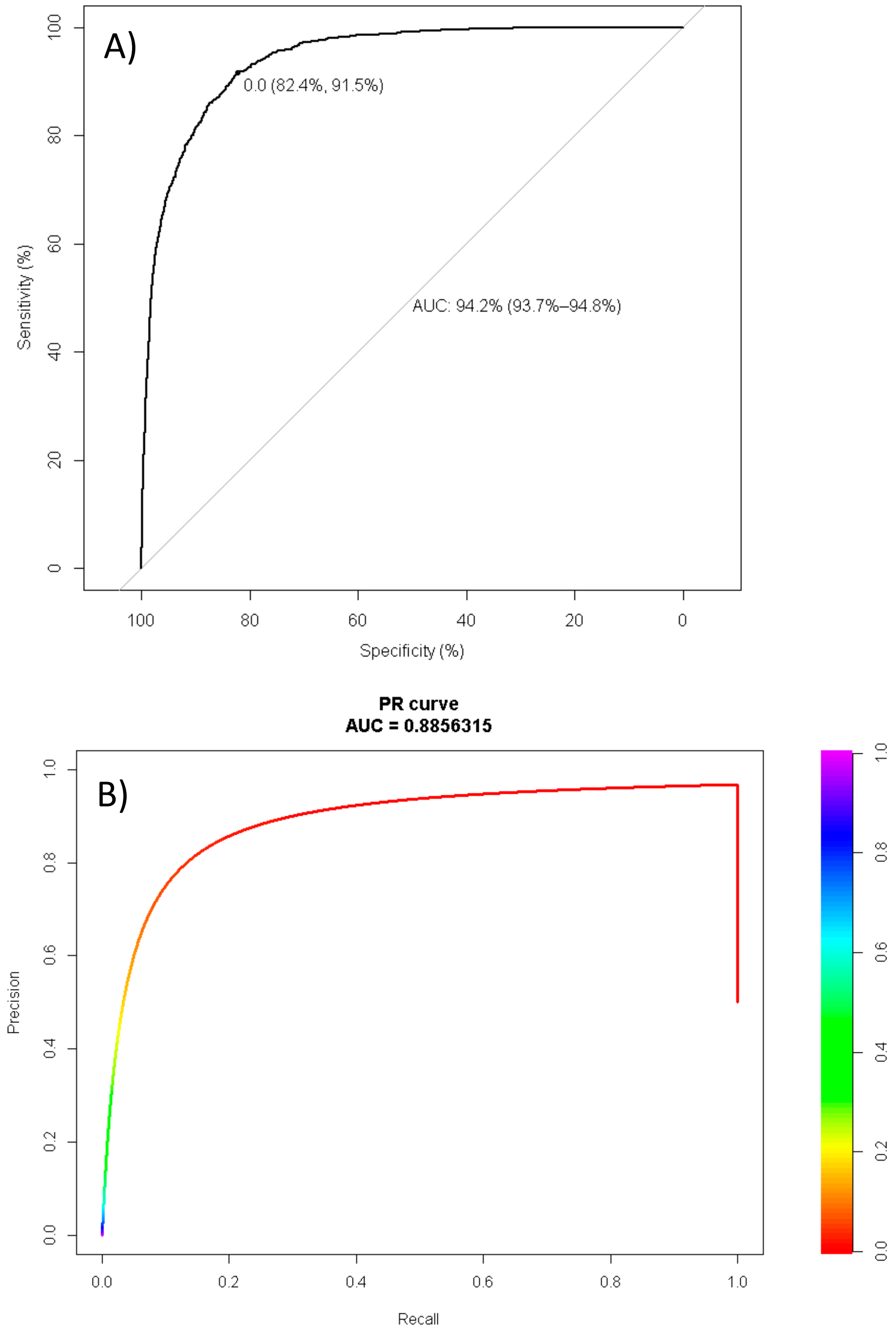

Figure S7. Predictive checking plots showing the total number of positive samplings observed (1205) and the predicted by the final model in 6000 iterations

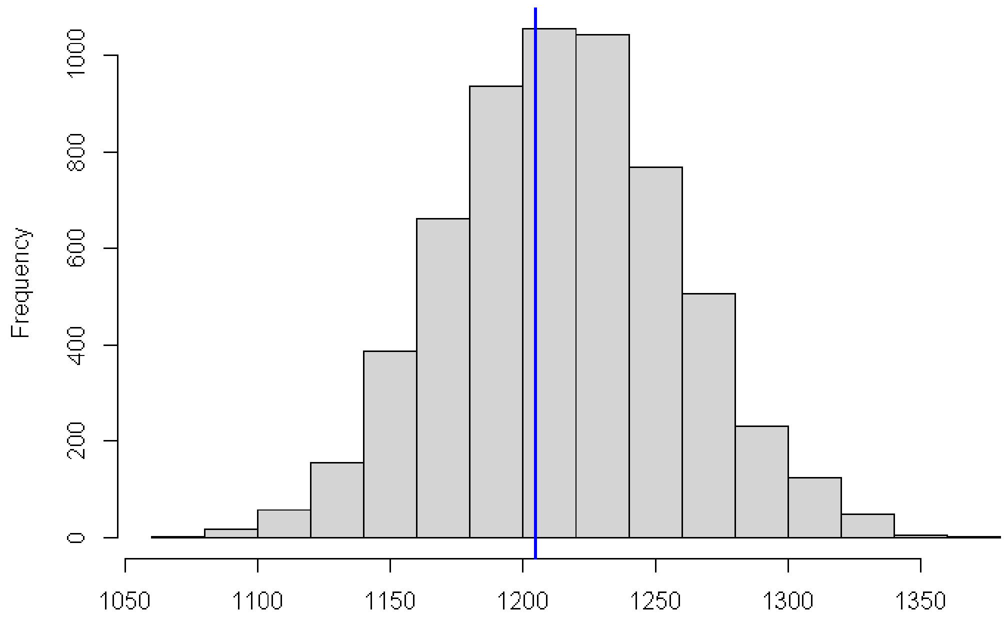

Figure S8. ROC (A) and precision-recall (B) curves based on the probability of a positive result in a sampling for *Salmonella* (target serovars)

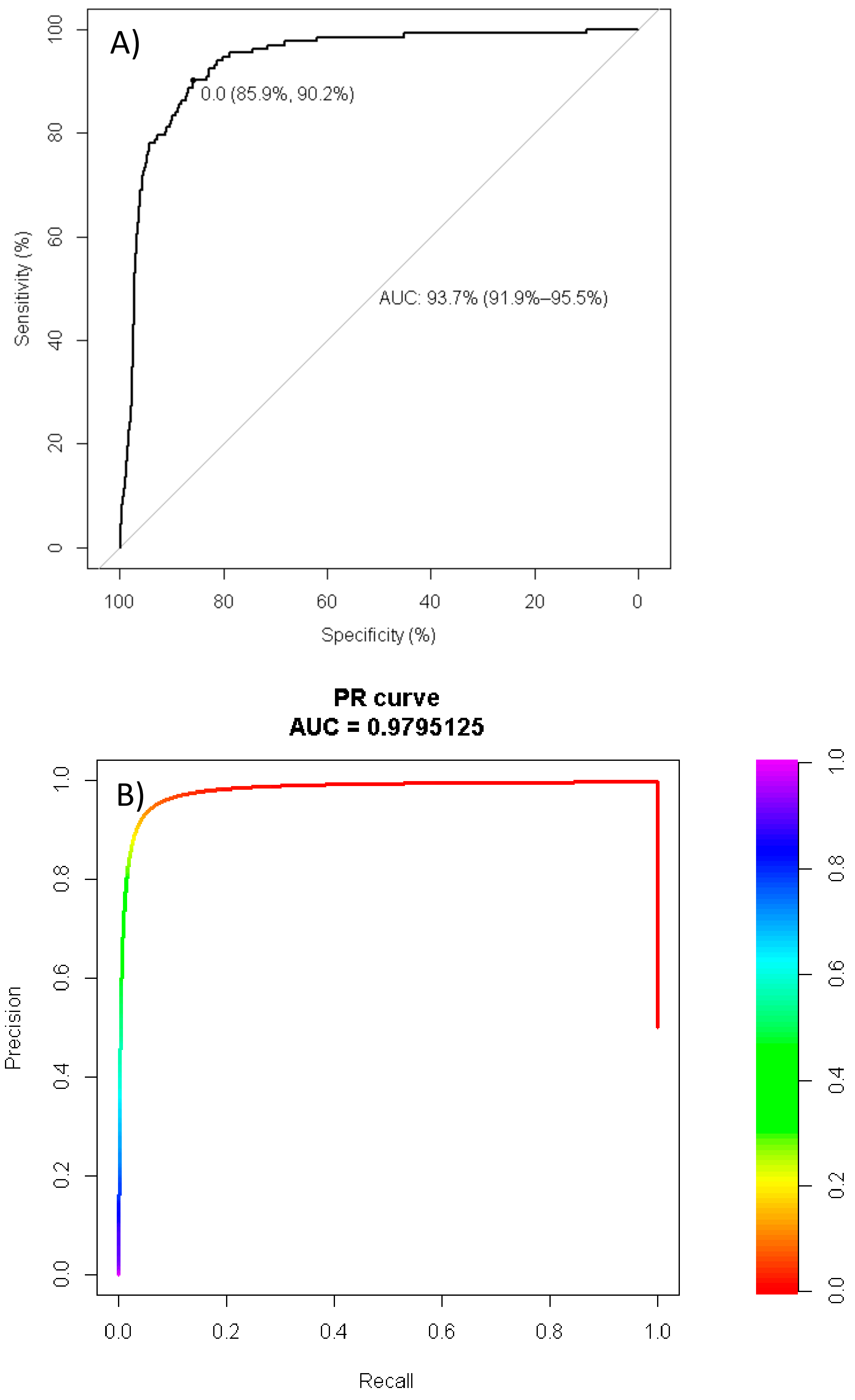

Supplement: Supplementary file 1 [file animals-13-03181-s001.zip › Supplementary Figures S1-S8_R1.pdf]
